# Association of obstructive sleep apnea and opioids use on adverse health outcomes: A population study of health administrative data

**DOI:** 10.1371/journal.pone.0269112

**Published:** 2022-06-28

**Authors:** Tetyana Kendzerska, Tara Gomes, Atul Malhotra, Andrea S. Gershon, Marcus Povitz, Daniel I. McIsaac, Shawn D. Aaron, Frances Chung, Gregory L. Bryson, Robert Talarico, Tahmid Ahmed, Michael Godbout, Peter Tanuseputro

**Affiliations:** 1 The Ottawa Hospital Research Institute/The Ottawa Hospital, Ottawa, Ontario, Canada; 2 Faculty of Medicine, Department of Medicine, University of Ottawa, Ontario, Canada; 3 ICES, Ottawa, Toronto, Ontario, Canada; 4 Leslie Dan Faculty of Pharmacy, University of Toronto, Ontario, Canada; 5 Li Ka Shing Knowledge Institute, Toronto, Ontario, Canada; 6 UC San Diego, San Diego, California, United States of America; 7 Sunnybrook Research Institute, Sunnybrook Health Sciences Centre, Toronto, Ontario, Canada; 8 Department of Medicine, University of Toronto, Ontario, Canada; 9 Department of Medicine, Cumming School of Medicine, University of Calgary, Calgary, Alberta, Canada; 10 Department of Anesthesiology and Pain Medicine, University of Ottawa, Ontario, Canada; 11 University Health Network, University of Toronto, Toronto, Ontario, Canada; 12 Bruyère Research Institute, Ottawa, Ontario, Canada; Brigham and Women’s Hospital and Harvard Medical School, UNITED STATES

## Abstract

**Rationale:**

Despite the high prevalence of obstructive sleep apnea (OSA) and concurrent use of opioid therapy, no large-scale population studies have investigated whether opioid use and pre-existing OSA may interact synergistically to increase the risk of adverse health consequences. To address this knowledge gap, we conducted a retrospective cohort study using provincial health administrative data to evaluate whether the combined presence of opioid use and OSA increases the risk of adverse health consequences, such as mortality, hospitalizations, and emergency department (ED) visits; and if it does, whether this co-occurrence has synergistic clinical relevance.

**Methods:**

We included all adults who underwent a diagnostic sleep study in Ontario, Canada, between 2013 and 2016. Individuals were considered exposed to opioids if they filled a prescription that overlapped with the date of their sleep study (Opioid+). Individuals with at least a 50% probability of having a diagnosis of moderate to severe OSA (OSA+) were identified using a previously externally validated case-ascertainment model. The primary outcome was all-cause mortality; secondary outcomes were all-cause or ischemic heart disease hospitalizations, all-cause ED visits, and motor vehicle collisions (MVC) requiring hospital or ED visit. We used multivariable Cox regression models to compare hazards between four mutually exclusive groups: (1) Opioid+ OSA+; (2) Opioid+ OSA-; (3) Opioid- OSA+, and (4) OSA- Opioid- (reference for comparison). Relative excess risks due to interaction (RERI) were calculated to test for additive interaction.

**Results:**

Of 300,663 adults who underwent a sleep study, 15,713 (5.2%) were considered as Opioid+ and 128,351 (42.7%) as OSA+. Over a median of two years, 6,223 (2.1%) died from any cause. Regardless of OSA status, opioid use at the date of the sleep study was associated with an increased hazard for all-cause mortality with the greatest hazard associated with Opioid+ OSA- (adjusted hazard ratio [aHR]: 1.75, 95% CI 1.57–1.94), but not Opioid+ OSA+ (aHR: 1.14, 95% CI 1.02–1.27) as hypothesized. Regardless of OSA status, opioid use at the date of the sleep study was associated with an increased hazard for all secondary outcomes. Opioid+ OSA+ was associated with the greatest hazards of all-cause hospitalizations (aHR 1.55, 95% CI 1.49–1.61) and MVC (aHR of 1.39; 95% CI 1.09–1.77); however, no statistically significant synergistic effects were observed.

**Conclusions:**

Adults referred for sleep disorder assessment who used opioids had a significantly increased hazard of adverse health outcomes than those who did not, regardless of whether they had a high probability of moderate to severe OSA. The use of opioids and OSA was associated with the greatest hazard of all-cause hospitalizations and MVC requiring hospital or ED visit. The interaction of opioids and OSA did not confer a synergistic risk for poor outcomes.

## Introduction

The ongoing opioid epidemic in North America [1] has led to an increased frequency of adverse opioid-related outcomes, such as higher rates of emergency department (ED) visits, hospitalization, and mortality [2, 3]. Although the current epidemic and adverse outcomes are no longer being driven by prescribed opioids for therapeutic use [4, 5], safe prescribing of opioids is still important [6]. Despite an overall decline in prescribed opioids over time, in some populations, prescriptions of long-term opioids increased as well as rates of a high-dose opioid use [7, 8]; thus, increasing the risk of complications associated with opioids, including altered sleep architecture, sleep quality, respiratory function during sleep, and increased daytime tiredness [9]. Nonfatal opioid-related outcomes, such as ED visits and hospitalizations, have been reported, even when opioids were used as directed [10]. Recent data also suggest the potential harm of commonly used opioids among individuals with cardiovascular disease [11]. Therefore, it is essential to understand the factors contributing to such outcomes, particularly in sub-populations with a higher risk.

Obstructive sleep apnea (OSA) is the most common reason for referral for sleep clinic assessment, and is one of the most prevalent sleep disordered breathing, with an estimated global prevalence of nearly one billion people [12]. OSA is characterized by repeated episodes of upper airway obstruction during sleep and requires an overnight sleep study for the diagnosis [13]. Individuals with untreated OSA have poorer quality of life, increased cardiovascular and metabolic risk, and make greater and more costly use of the healthcare system than the general population [14–19]. Societal impacts of OSA through daytime sleepiness include an increased risk of motor vehicle collisions (MVC), workplace accidents, and lost productivity [20–23].

It is estimated that 36% to 85% of individuals taking opioids may have sleep disordered breathing, including OSA [24–27]. We demonstrated a higher prevalence of chronic opioid use with a large proportion on long-acting opioids and higher opioid dosages among adults referred for a sleep disorder assessment than the general population [28]. This high prevalence is important because plausible pathogenetic mechanisms exist suggesting that opioids may not only be a risk factor for OSA, but may also adversely impact respiratory function among individuals with OSA [27, 29] through a decrease in airway muscle tone, the output of the respiratory pacemaker, and central respiratory drive [30]. However, the current evidence on the effects of opioids on OSA is inconsistent [31–33]. Previous studies suggested that only a subset of OSA patients may be at increased risk for opioid-induced ventilatory compromise. Despite the high prevalence of OSA and concurrent use of opioid therapy, no large-scale population studies have investigated whether opioid use and pre-existing OSA may interact synergistically to increase the risk of adverse health consequences [31].

To address this knowledge gap, we evaluated whether the combined presence of opioid use and OSA increases the risk of adverse health consequences, such as mortality, hospitalizations, and ED visits; and if it does, whether this co-occurrence has synergistic clinical relevance.

## Methods

### Study design

We conducted a retrospective longitudinal population-based cohort study utilizing provincial health administrative data in Ontario, the most populous province in Canada. The use of data in this project was authorized under section 45 of Ontario’s Personal Health Information Protection Act, which does not require review by a Research Ethics Board.

#### Data sources

ICES is an independent, non-profit research institute whose legal status under Ontario’s health information privacy law allows it to collect and analyze health care and demographic data, without consent, for health system evaluation and improvement. Since 1991, high-quality administrative databases [34] on publicly funded services provided by physicians and hospitals for all Ontario residents are housed at ICES, including individual-level information on outpatient and outpatient visits, including procedures [35]. A description of databases is available at https://datadictionary.ices.on.ca/Applications/DataDictionary/. In this study, we used the Registered Persons Database (RPDB); Same Day Surgery Database (SDS); the National Ambulatory Care Registry System (NACRS); the Ontario Health Insurance Plan (OHIP) database; the Canadian Census; the ICES Physician Database (IPDB), and ICES-derived disease-specific databases. We also used the Narcotics Monitoring System (NMS) database to capture data on dispensed prescriptions for controlled substances and other monitored drugs such as opioids, benzodiazepines/zolpidem, barbiturates, and stimulants since July 1, 2012. Furthermore, for all insured Ontario residents who underwent an overnight sleep study in a sleep laboratory setting and have been diagnosed with OSA by a sleep physician registered with the Assistive Devices Program (ADP), partial funding is provided for positive airway pressure (PAP) systems, recommended treatment for individuals with moderate to severe OSA [36–42], and documented in the ADP database [43]. Funding eligibility does not depend on OSA severity or PAP therapy use. Details on eligibility criteria are available at www.ontario.ca/page/respiratory-equipment-and-supplies and www.ontvep.ca/about-us. These databases were linked using unique encoded identifiers at ICES.

#### Study population

All adults aged 18 years and older who underwent a diagnostic sleep study from ***July 2013 to June 2016*** were included. We chose this time to ensure (i) a one-year lookback window to identify opioid users through the NMS and (ii) more than one year of follow-up for each included subject. The ***index date*** was the date of the diagnostic sleep study. Each included individual was followed forward from the index date until the end of the follow-up period (***March 31*, *2018***), emigration from Ontario, or until death, whichever came first.

We excluded individuals who: (1) were in long-term care [44] or received palliative care [45] in the year prior to the index date; (2) were already on PAP at the index date or underwent a therapeutic sleep study during the last five years preceding the index date; or (3) at the index date, were taking opioids which are rarely used and/or with not well-defined morphine equivalencies, such as intranasal, injectable, or rectal suppositories. Details on definitions and exclusion criteria are also provided in the **Data Supplement (S1 Table)**.

### Primary exposures

#### Case-ascertainment model to identify individuals with moderate to severe OSA

We used the previously validated case-ascertainment model against a diagnostic sleep study (gold standard) to identify individuals with moderate to severe OSA [46]. This model contained six variables in relation to an index sleep study: an outpatient visit for OSA from a specialist physician, a repeated sleep study and a PAP treatment claim within 1 year of the index sleep study, patient sex and age at the index sleep study and hospitalizations with hypertension in the last 3 years prior to the sleep study [46]. On the external cohort, this definition yielded a sensitivity of 59% (95% CI: 58–60), specificity of 87% (95% CI: 0.87–0.88), a positive predictive value of 0.79 (95% CI: 0.78–0.80) and negative predictive value of 0.73 (95% CI: 0.72–0.74) to identify individuals ***with an estimated probability of 0*.*5 or greater*** of moderate to severe OSA [46]. For the ***primary analysis***, all individuals with an estimated probability of 0.5 or greater using the case-ascertainment models were classified as having OSA. For the ***secondary analyses***, the probability of moderate to severe OSA was considered as a continuous variable ranging between 0 and 1.

#### Opioid use at the index date

All opioids dispensed between ***July 2012 and March 2018*** were identified through the NMS database, including oral formulations of morphine, codeine, oxycodone, meperidine, hydromorphone, pentazocine, tramadol, tapentadol, and opium, as well as transdermal fentanyl, and buprenorphine patches.

Individuals were considered *on opioids at the index date* if an opioid prescription duration overlapped the index date. We calculated the average morphine equivalent daily dose on the index date based on the number of tablets dispensed, the strength of the medications and the number of days’ supply.

### Outcomes

Our *primary outcome* was all-cause mortality. As secondary outcomes, we considered any hospital admission or hospitalization for ischemic heart disease (IHD) [47] any ED visit, and MVC requiring hospitalization or ED visits in which an individual was a driver of the motor vehicle [48].

### Risk adjustment covariates

The following factors were considered as potential confounders or risk factors in the statistical model prior or at the index date: (i) demographics: age, sex, place of residence, and neighborhood income quintile as a measure of socioeconomic status [49]; (ii) receipt of benzodiazepines within the year prior to the index date [50]; (iii) separate prevalent comorbidities (hypertension, diabetes, psychiatric comorbidities, arthritis, asthma, COPD [including severe COPD which may require an opioid prescription], cancer, cardiovascular, liver and kidney diseases); (iv) Charlson comorbidity index, a weighted index of comorbidities for predicting mortality [51]; (v) any outpatient or inpatient surgical intervention in the last year; (vi) substance use disorder [52] (including opioid use) and neuromuscular disorder in the last five years; and (vii) number of the primary care office visit in the last year prior to the index date as a measure of the prior health care exposure. Where relevant, we used validated algorithms to ascertain specific conditions [53–60]. Details on definitions and diagnostic codes used are provided in the **Data Supplement (S1 Table)**.

### Analyses

Descriptive statistics were used to characterize the study population overall and by exposures at the index date. Unadjusted Kaplan-Meier survival curves for the primary outcome and all-cause mortality were plotted by exposure status and compared between groups using the log-rank test. For secondary outcomes, we estimated incidence with the cumulative incidence function, which accounts for competing risks [61], and compared between groups using Gray’s test.

#### Primary analyses

To quantify interaction on the additive scale [62, 63], we followed a recommended analytic approach, which involves constructing one categorical variable with four levels that combines two dichotomous determinants (***primary exposures***). Specifically, to investigate the combined effect of opioid use and moderate to severe OSA on outcomes of interest, using univariate and multivariable Cox regression model with all covariates defined above, hazards of each outcome, separately, were compared between four mutually exclusive groups: (1) individuals with at least 50% probability of moderate to severe OSA who were exposed to opioids (Opioid+ OSA+); (2) individuals with less than 50% probability of moderate to severe OSA who were exposed to opioids (Opioid+ OSA–); (3) individuals with at least 50% probability of moderate to severe OSA who are not exposed to opioids (Opioid–OSA+), and (4) individuals with less than 50% probability of moderate to severe OSA who are not exposed to opioids (Opioid–OSA–; reference group). Caused-specific multivariable Cox regression models to adjust for all-cause mortality as a competing event were used to investigate the relationship between four groups of interest and secondary outcomes.

Based on obtained adjusted hazard ratios (aHRs), we calculated three measures of biological interaction on the additive scale: (i) the relative excess risk due to interaction (RERI), (ii) the attributable proportion due to interaction (AP), and (iii) the synergy index (S) (**S1 Text**) [62, 64]. A RERI of zero indicates *no interaction*, a RERI greater than zero indicates a *synergetic interaction*, and a RERI less than zero indicates a *negative interaction*.

#### Secondary analyses

To check the robustness of our findings, we included a statistical interaction term between opioid prescription on the index date and probability of moderate to severe OSA (range between 0 and 1). To account for nonlinearity, we transformed the continuous probability of moderate to severe OSA via a 5-knot restricted cubic spline (knot placements: 5th, 27.5th, 50th, 72.5th, 95th percentiles). Adjusted HRs and 95% CI comparing Opioid+ vs. Opioid- were computed and visualized at incremental OSA probability thresholds (from 0 to 1 by 0.1 units) [65, 66].

Finally, we tested the effect of prescribed opioids and a high probability of OSA as separate independent variables in the statistical model adjusting for confounders.

All statistical analyses were performed in the secure environment at ICES following Ontario privacy standards using SAS 9.3 (SAS Institute Inc., Cary, NC).

## Results

### Description of population characteristics

We found that in Ontario between 2013 and 2016, a total of 300,663 adults (median age of 51 years; 169,309 [56.3%] male; 55,575 [18.5%] reside at the lowest neighbourhood income quintile) were referred to a sleep clinic for a sleep disorder assessment, underwent an initial diagnostic sleep study and met our inclusion criteria (**Table 1**).

**Table 1:** Baseline characteristics at the date of the diagnostic sleep study for the entire cohort and by active opioid prescription (being on opioids at the date of the diagnostic sleep study [index date]) and at least a 50% probability of moderate to severe obstructive sleep apnea (OSA)*.

Characteristics			Opioid- OSA-	Opioid- OSA+	Opioid+ OSA-	Opioid+ OSA+	TOTAL
			N = 163,657	N = 121,293	N = 8,655	N = 7,058	N = 300,663
***Demographics at the index date***							
**Age, years; Median (IQR)**			48 (37–58)	55 (45–64)	52 (43–61)	57 (50–65)	51 (41–61)
**Sex: Male**			78,767 (48.1)	82,853 (68.3)	3,530 (40.8)	4,159 (58.9)	169,309 (56.3)
**Neighbourhood Income Quintile**		**1 (lowest)**	29,900 (18.3)	21,392 (17.6)	2,391 (27.6)	1,892 (26.8)	55,575 (18.5)
		**2**	32,409 (19.8)	24,163 (19.9)	1,973 (22.8)	1,601 (22.7)	60,146 (20.0)
		**3**	32,911 (20.1)	24,898 (20.5)	1,676 (19.4)	1,383 (19.6)	60,868 (20.2)
		**4**	33,664 (20.6)	25,044 (20.6)	1,441 (16.6)	1,165 (16.5)	61,314 (20.4)
		**5 (highest)**	34,389 (21.0)	25,574 (21.1)	1,149 (13.3)	1,007 (14.3)	62,119 (20.7)
**Rurality: Yes**			15,849 (9.7)	13,944 (11.5)	1,263 (14.6)	1,181 (16.7)	32,237 (10.7)
***Comorbidities*, *primary health care exposure*, *surgical interventions and controlled substances use in the last year***							
**Charlson Comorbidity Index (CCI)**	**None (CCI score = 0)**		157,851 (96.5)	113,930 (93.9)	7,666 (88.6)	6,014 (85.2)	285,461 (94.9)
	**Low (CCI score = 1)**		2,604 (1.6)	3,075 (2.5)	457 (5.3)	412 (5.8)	6,548 (2.2)
	**Moderate (CCI score = 2)**		1,838 (1.1)	2,551 (2.1)	280 (3.2)	323 (4.6)	4,992 (1.7)
	**High (CCI score ≥ 3)**		1,364 (0.8)	1,737 (1.4)	252 (2.9)	309 (4.4)	3,662 (1.2)
**Number of Primary Care Visits, Median (IQR)**			4 (2–8)	4 (2–8)	9 (5–15)	8 (5–13)	5 (2–8)
**Surgery/Intervention Indicator**			5,362 (3.3)	5,639 (4.6)	634 (7.3)	662 (9.4)	12,297 (4.1)
**Benzodiazepine Dispensed**			24,073 (14.7)	15,299 (12.6)	3,626 (41.9)	2,527 (35.8)	45,525 (15.1)
**Cannabinoids Dispensed**			481 (0.3)	250 (0.2)	445 (5.1)	219 (3.1)	1,395 (0.5)
**Stimulants Dispensed**			2,925 (1.8)	1,181 (1.0)	251 (2.9)	132 (1.9)	4,489 (1.5)
***Prior comorbidities***							
**Chronic heart failure**			4,382 (2.7)	6,309 (5.2)	554 (6.4)	687 (9.7)	11,932 (4.0)
**Chronic obstructive pulmonary disease**			14,684 (9.0)	16,079 (13.3)	2,278 (26.3)	2,105 (29.8)	35,146 (11.7)
**Coronary artery disease**			13,202 (8.1)	17,149 (14.1)	1,246 (14.4)	1,507 (21.4)	33,104 (11.0)
**Diabetes**			23,581 (14.4)	28,227 (23.3)	2,292 (26.5)	2,431 (34.4)	56,531 (18.8)
**Hypertension**			51,881 (31.7)	60,104 (49.6)	4,001 (46.2)	4,452 (63.1)	120,438 (40.1)
**Non-psychotic Mood and Anxiety Disorders**			45,667 (27.9)	25,960 (21.4)	3,885 (44.9)	2,595 (36.8)	78,107 (26.0)
**Cancer**			7,618 (4.7)	8,434 (7.0)	573 (6.6)	632 (9.0)	17,257 (5.7)

*Unless otherwise specified, results were presented as numbers and percentages per column in a bracket.

CCI, Charlson Comorbidity Index; IQR, interquartile range; OSA, obstructive sleep apnea

As exposures of interest, 15,713 (5.2%) were active opioid users and 128,351 (42.7%) had at least 50% probability of moderate to severe OSA. Among active opioid users, 5,699 (36.3%) were on long-acting opioids and 2,458 (15.6%) were on total daily dose ≥200 mg of morphine or equivalent [29]. Most often prescribed opioids were codeine (4,323 [27.5%]), hydromorphone (2,155 [13.7%]), and oxycodone (1,620 [10.3%]).

Over a median follow-up of 22.4 months (IQR: 7.8–34.6), 2.1% of the cohort (6,223/300,663) died from any cause, 21.0% (63,208/300,663) were hospitalized for any cause, 2,8% (8,549/300,663) had an IHD-related hospitalization, 55.5% (166,997/300,663) visited an ED for any cause, and 0.6% (1,942/300,663) were hospitalized or went to ED for MVC.

### The combined effect of opioid use and OSA

Characteristics of individuals by four levels of exposures are presented in **Table 1**. 7,058 (2.3%) were considered as Opioid+ OSA+; 8,655 (2.9%) as Opioid+ OSA–; 121,293 (40.3%) as Opioid–OSA+; and 163,657 (54.4%) as Opioid–OSA–. As compared to other groups, individuals in the Opioid+ OSA+ group were more likely to be older, reside in a rural area, have a higher Charlson comorbidity index and surgical interventions in the last year, and have prevalent coronary artery disease, COPD, diabetes, and hypertension.

**Fig 1:**
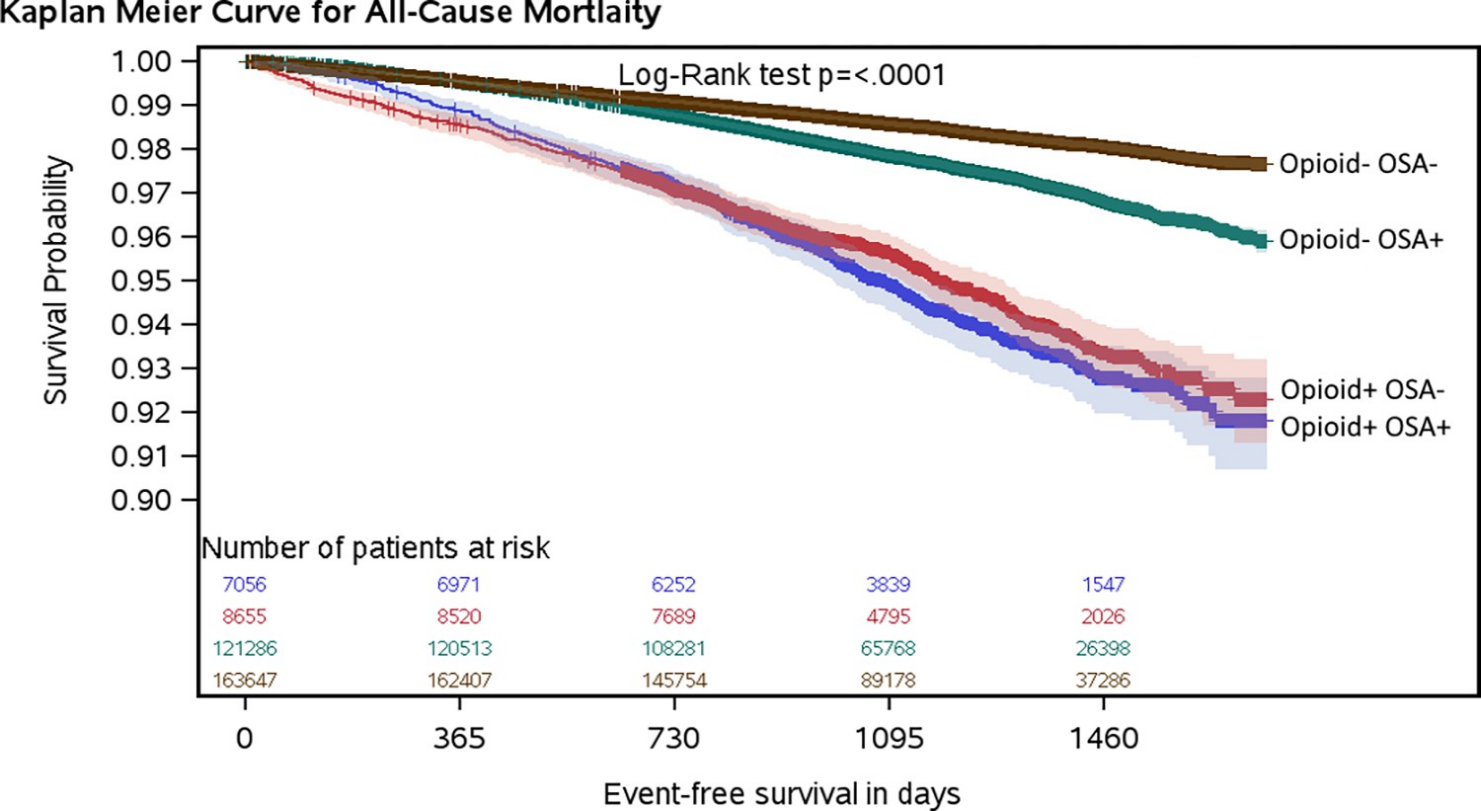
Unadjusted Kaplan-Meier survival curves by exposures of interest: at least a 50% probability of moderate to severe obstructive sleep apnea (OSA+) and active opioid use (Opioid +). The numbers at risk are presented above the x-axis: from top to bottom, Opioid+ OSA+; Opioid+ OSA-; Opioid- OSA+; Opioid- OSA-.

**Fig 2:**
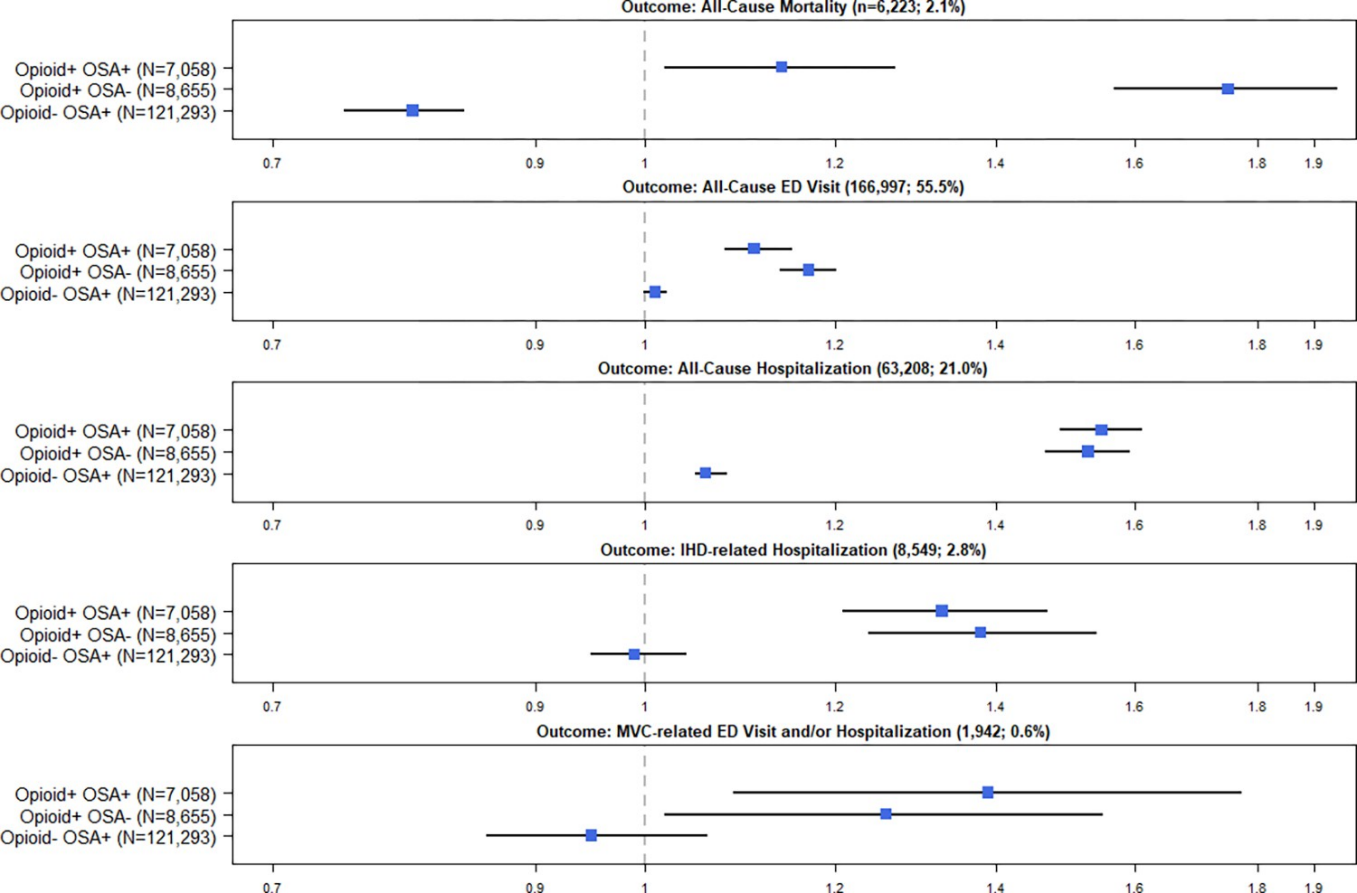
The effect of the presence of opioid use (Opioid +) and at least a 50% probability of moderate to severe obstructive sleep apnea (OSA+) on outcomes compared to a reference group (Opioid-OSA-) controlling for confounders. Effects expressed as adjusted hazard ratios and 95% confidence intervals.

For the primary outcome, unadjusted Kaplan-Meier survival curves (**Fig 1**) and univariate Cox regression (**Table 2**) demonstrated worse survival associated with the combined presence of opioid use and OSA in a hypothesized order: Opioid+ OSA+, Opioid+ OSA–, Opioid–OSA+, Opioid–OSA–. Controlling for confounders, opioid use at the date of the sleep study was associated with an increased hazard for all-cause mortality, regardless of OSA status. However, the highest hazard was associated with Opioid+ OSA- (adjusted hazard ratio [aHR]: 1.75, 95% CI 1.57–1.94), but not Opioid+ OSA+ (aHR: 1.14, 95% CI 1.02–1.27) as hypothesized. The synergistic effect was not confirmed (**Table 2; Fig 2**). Contrastingly, a possible protective effect of OSA was noted; aHR for Opioid- OSA+ vs. Opioid- OSA- of 0.80 (95% CI: 0.75–0.84) with RERI suggestive of a negative interaction: -0.41 (-0.62 to -0.20).

**Table 2:** Hazard ratios of all-cause mortality, all-cause emergency department (ED) visits, ischemic heart disease (IHD)-related hospitalizations, motor vehicle collision (MVC) related ED visits and/or hospitalizations and RERI, AP, synergy index, multiplicative measure of Interaction by four levels of exposures.

Outcomes Exposures	All-cause Mortality (N = 6,204)	All-cause ED Visit (N = 166,997)	All-cause Hospitalization (N = 63,208)	IHD-related Hospitalization (N = 8,549)	MVC related ED Visit or Hospitalization (N = 1,942)
**Unadjusted Hazard Ratios (95% Confidence Interval)**					
**Opioid+ OSA+**	3.59 (3.23–4.00)	1.56 (1.51–1.60)	2.72 (2.62–2.83)	3.76 (3.42–4.13)	1.63 (1.30–2.05)
**Opioid+ OSA-**	3.31 (2.99–3.67)	1.67 (1.63–1.72)	2.34 (2.26–2.43)	2.35 (2.12–2.61)	1.75 (1.43–2.14)
**Opioid- OSA+**	1.55 (1.47–1.64)	0.99 (0.98–1.00)	1.26 (1.24–1.28)	1.88 (1.80–1.97)	0.78 (0.70–0.85)
**Opioid- OSA-**	Reference	Reference	Reference	Reference	Reference
**Measures of Interactions** *					
**RERI**	-0.27 (-0.75 to 0.21)	-0.11 (-0.17 to -0.05)	0.13 (0.00 to 0.26)	0.52 (0.12 to 0.93)	0.11 (-0.39 to 0.61)
**AP**	-0.08 (-0.22 to 0.06)	-0.07 (-0.11 to -0.03)	0.05 (0.00 to 0.09)	0.14 (0.04 to 0.24)	0.07 (-0.23 to 0.36)
**Synergy Index**	0.90 (0.76 to 1.08)	0.83 (0.75 to 0.92)	1.08 (1.00 to 1.17)	1.23 (1.05 to 1.45)	1.21 (0.50 to 2.91)
**Adjusted# Hazard Ratios (95% Confidence Interval)**					
**Opioid+ OSA+**	1.14 (1.02–1.27)	1.11 (1.08–1.15)	1.55 (1.49–1.61)	1.33 (1.21–1.47)	1.39 (1.09–1.77)
**Opioid+ OSA-**	1.75 (1.57–1.94)	1.17 (1.14–1.20)	1.53 (1.47–1.59)	1.38 (1.24–1.54)	1.26 (1.02–1.55)
**Opioid- OSA+**	0.80 (0.75–0.84)	1.01 (1.00–1.02)	1.06 (1.05–1.08)	0.99 (0.95–1.04)	0.95 (0.86–1.06)
**Opioid- OSA-**	Reference	Reference	Reference	Reference	Reference
**Measures of Interactions** *					
**RERI**	-0.41 (-0.62 to -0.20)	-0.07 (-0.11 to -0.03)	-0.05 (-0.13 to 0.03)	-0.04 (-0.23 to 0.14)	0.18 (-0.23 to 0.58)
**AP**	-0.36 (-0.56 to -0.16)	-0.06 (-0.10 to -0.02)	-0.03 (-0.08 to 0.02)	-0.03 (-0.17 to 0.11)	0.13 (-0.14 to 0.40)
**Synergy Index**	0.25 (0.10 to 0.62)	0.62 (0.45 to 0.86)	0.92 (0.80 to 1.05)	0.88 (0.52 to 1.48)	1.86 (0.41 to 8.50)
**Adjusted# Hazard Ratios (95% Confidence Interval): each exposure was considered as a separate independent variable**					
**Opioid+ vs. Opioid-**	1.58 (1.46–1.71)	1.14 (1.12–1.16)	1.49 (1.45–1.53)	1.36 (1.26–1.46)	1.34 (1.13–1.58)
**OSA+ vs. OSA-**	0.78 (0.74–0.82)	1.01 (1.00–1.02)	1.06 (1.04–1.08)	0.99 (0.95–1.03)	0.97 (0.88–1.07)

*Relative Excess Risk of Interaction (RERI) can range from − infinity to + infinity. RERI = 0 means no interaction or exact additivity; RERI > 0 means positive interaction or more than additivity; RERI < 0 means negative interaction or less than additivity. For example, RERI [62] was calculated using the following formula: HR for the combined estimated effect (Opioid + OSA+) minus the effects (HR) of each exposure considered individually (Opioid+ OSA–, Opioid–OSA+) plus one (reference) [62].

Attributable Proportion (AP) can range from −1 to +1. AP = 0 means no interaction or exactly additivity; AP > 0 means positive interaction or more than additivity; AP < 0 means negative interaction or less than additivity

Synergy Index (S) can range from 0 to infinity. S = 1 means no interaction or exactly additivity; S > 1 means positive interaction or more than additivity; S < 1 means negative interaction or less than additivity

# Hazard Ratios are adjusted for: baseline demographics (age, sex, income quintiles), location of residence (rural vs. urban), benzodiazepine dispensed within last year, alcohol use disorder, cancer, separate prevalent comorbidities (heart diseases, COPD, diabetes, hypertension, mental health conditions, osteoarthritis, neuromuscular diseases), number of primary care visits, Charlson Comorbidity Index, and surgical interventions in the last year.

AP, Attributable Proportion; ED, emergency department; IHD, ischemic heart disease; MVC, motor vehicle collision; OSA, obstructive sleep apnea; RERI, Relative Excess Risk of Interaction

For secondary outcomes, in univariate analysis, the highest hazards associated with Opioid+ OSA+ were noted for all-cause and IHD-related hospitalizations, with a potential synergetic effect noticed for all-cause and IHD-related hospitalizations, as well as MVC-related ED visit or hospitalization (**S1–S4 Figs; Table 2**). Controlling for confounders, regardless of OSA status, opioid use at the index date was associated with an increased hazard for all secondary outcomes. The highest hazard associated with Opioid+ OSA+ was noted for all-cause hospitalization (aHR 1.55, 95% CI 1.49–1.61) and MVC-related ED visit or hospitalization (aHR of 1.39; 95% CI 1.09–1.77). A potential but not significant synergetic effect was noted for MVC-related outcome only: RERI = 0.18 (-0.23 to 0.58) (**Table 2; Fig 2**).

Details on the effect of other covariates are presented in **S2 Table**. Controlling for other covariates, living in lower neighbourhood income or rural area, alcohol use disorder/intoxication, neuromuscular disease, and prevalent coronary artery disease were significantly associated with all outcomes of interest. Benzodiazepine use at any time in the last year prior to the index date and higher Charlson Comorbidity Index were both significantly associated with all-cause mortality and ED visits and with all-cause and IHD-related hospitalizations. The effect of age, sex and other separate comorbidities differed depending on the outcome.

We confirmed our findings in the secondary analysis (**S5–S9 Figs; S3 Table**). Specifically, prescribed opioids were associated with increased hazards of outcomes at any probability of OSA. For all outcomes but not all-cause mortality, hazard ratios for the opioid effect remained relatively similar across different probabilities of OSA. An increase in hazards was noted for all-cause mortality at lower OSA probability (<0.4).

## Discussion

Despite the high prevalence of OSA and concurrent use of opioids, no large-scale population studies have investigated whether opioid use and pre-existing OSA may interact synergistically to increase the risk of adverse health consequences. In this population-based study among adults who underwent a diagnostic sleep study, individuals who used opioids had a significantly increased risk of all-cause mortality, hospitalizations, ED visits, IHD-related hospitalization and MVC related ED visit or hospitalization, compared to those who did not, regardless of whether they had a high probability of OSA. The combined effect of active opioid use and high probability of OSA was associated with the greatest hazard of all-cause hospitalization, and hospitalization or ED visit for MVC, but not with all-cause mortality, all-cause ED visits, and IHD-related hospitalization. The interaction of opioids and OSA did not confer a synergistic risk for poor outcomes. Although we did not confirm the hypothesis on the synergistic clinical relevance on the combined effect of opioid use and OSA, importantly, we demonstrated that in the adult population with a high pre-test probability of sleep disordered breathing, prescribed opioid use was significantly associated with all outcomes of interest, suggesting safe prescribing of opioids is still vital, especially in this population.

While a higher prevalence of moderate to severe OSA was expected among individuals referred for a sleep disorder assessment (43% in our study population vs. 18% of the estimated population prevalence [12]), in our study, the proportion of individuals with a high probability of moderate to severe OSA was not considerably higher among active opioid users (7,058/15,713; 45%). This confirms the results of our previous study when the pooled prevalence of sleep disordered breathing among individuals with chronic pain on opioid therapy was not significantly different compared to those not on opioid therapy with or without pain [26]. Further, in our recent systematic review and meta-analysis, we did not demonstrate a significant relationship between opioid use and the severity of OSA [33].

Opioid use may affect breathing during sleep through a decrease in airway muscle tone, the output of the respiratory pacemaker, and/or central respiratory drive [30]. Individuals with OSA may be more vulnerable to the respiratory depressant effects of opioids compared to those without OSA [67], which may lead to adverse health outcomes in this population. Conversely, among individuals with OSA, the sedating effect of opioids may stabilize airway patency and breathing [68]. Acute administration of 40 mg controlled-release oral morphine did not worsen OSA [69], or systematically impair airway collapsibility, pharyngeal muscle responsiveness or the arousal threshold among individuals with moderate to severe OSA [70]. A large inter-individual variability noted in those studies suggests that only a subset of individuals with OSA with specific clinical phenotype and genotype may be at increased risk for opioid-induced ventilatory compromise, and consequently, contribute to the synergetic clinical relevance [69, 70]. These findings may explain why we have not found greater hazards associated with the combined effect of OSA and opioid use for all outcomes, and why we have not confirmed the synergetic effect. The potential survival advantage of OSA was previously hypothesized through ischemic preconditioning resulting from the nocturnal cycles of hypoxia-reoxygenation as a possible explanation for the age decline relative mortality in sleep apnea [71, 72], and the morbidity-mortality paradox of obesity [73]. However, we could not exclude the risk of the statistical model overcontrolling, misclassification bias and unmeasured confounding (for example, information on body mass index was not available) impacting our results. Another explanation is confounding by PAP treatment, as the risk of death from respiratory suppression is lowered by PAP therapy [74]. However, at the same time, we cannot exclude a healthy user effect, i.e., those who are in better health claimed PAP and live longer, or a survival bias as some individuals may not live long enough to get PAP therapy. Finally, two years of follow-up for some individuals may be inadequate for all-cause mortality as an outcome.

While prescribed opioid use has been shown to be associated with increased mortality, hospitalizations, and ED visits [3, 4], the effect on cardiovascular outcomes remains controversial [11]. In our study, we demonstrated an increased hazard of IHD-hospitalizations associated with prescribed opioids regardless of OSA status. The potential for interactions of opioids for pain management and medications used to treat cardiovascular disease on outcomes has recently been investigated [11]. For example, it has been shown that morphine may delay clopidogrel absorption, decrease plasma levels of clopidogrel active metabolite, and delay and diminish its effects, which can lead to treatment failure among susceptible individuals [75]. Use of morphine either alone or in combination with nitroglycerin for individuals presenting with non–ST-segment elevation acute coronary syndromes has been shown to be associated with higher mortality [76]. Current limited evidence along with our findings suggests potential harm for these individuals, and the need for more evidence-based research on the effect of opioids among individuals with cardiovascular disease.

Despite that opioids, as centrally-acting medications, may interfere with the ability to drive a motor vehicle safely [77, 78], studies evaluating the impact of opioids on driving-related psychomotor skills report contradictory findings. This is likely due to heterogeneity in the study design, assessment tools, and diverse study populations. A recent systematic review did not identify impaired simulated driving performance when individuals with chronic pain or chronic breathlessness took regular therapeutic opioid agonists for symptom control [79]. The hazard of an ED visit for injuries related to an MVC was shown to be similar for opioid and nonsteroidal anti-inflammatory drug recipients after initiation of analgesic therapy [80]. Our study demonstrated an increased hazard of MVC-related outcomes associated with prescribed opioids among individuals referred for sleep disorder assessment. Importantly, we also showed a higher hazard associated with combined OSA and opioid use, as well as a potential synergetic effect. The 2021 Canadian Council of Motor Transport Administrators (CCMTA) recognizes severe OSA as a factor that increases collision risk and suggests that a driver is eligible for a licence “if [she or he] has untreated obstructive sleep apnea with an apnea-hypopnea index (AHI) < 30 and does not admit to daytime sleepiness” [81]. If our findings are confirmed in future studies with known information on daytime sleepiness, a lower AHI threshold needs to be considered among individuals with both OSA and prescribed opioids, even in the absence of daytime sleepiness.

Notably, in addition to comorbidities, living in lower neighbourhood income or rural area, alcohol use disorder/intoxication, and benzodiazepine prescription were associated with adverse health outcomes in our study, confirming the results from previous studies [82–85] and identifying vulnerable populations.

Our study has several strengths, including the use of real-world, population-level data, with nearly complete follow-up and access to high quality definitions of outcomes and validated definition of OSA, which allow us to examine the combined effect of opioid use and the high probability of OSA on adverse health outcomes on the population level.

It is important to mention several limitations associated with our health administrative data-based study, such as predisposition for misclassification bias and unmeasured confounding, given that health administrative data are not primarily collected for research purposes. However, validation studies demonstrate that the information on which our main measures rely (i.e., physician billing data, vital statistics, healthcare resource use, prescription data) are valid and accurate [34]. Next, since the Narcotics Monitoring System does not capture opioids provided in prisons or hospitals, we were also unable to account for illicit opioid exposure. However, these misclassification rates are likely low among a primarily community-based sample. While sleep studies can be accurately identified from health administrative data given the current management of OSA in Ontario, information on the severity of OSA and level of daytime sleepiness was not available. To address this limitation, we have previously externally validated the definition for OSA used in this study [46]: Our case-ascertainment models for identifying moderate/severe OSA using health administrative data had relatively low sensitivity but high specificity and good discriminative ability. To address this limitation, we considered a probability of OSA as a continuous variable with a wide range of sensitivities and specificities (measurement properties for OSA probability thresholds from 0 to 1 by 0.1 units presented in **S1 Table** and were reported previously [46]. Misclassification of OSA status would likely bias our results towards a reduced difference between hazards associated with the combined presence of opioids use and OSA vs. opioid use only, and may prevent us from seeing the greater hazards associated with the combined effect. Our model performed poorly while including a measure of daytime sleepiness [46], highlighting the challenge of using health administrative data to identify OSA clinical subtypes due to high heterogeneity in clinical OSA presentation. We were also not able to identify individuals with central sleep apnea (CSA), with an estimated prevalence in chronic opioid users of 24% [86]. However, there is no direct evidence of any major clinical consequence from CSA among chronic opioid users [87]. Finally, although the information on PAP therapy use was not available in our study, it is usually poor in the general population [88], and specifically among individuals with OSA and prescribed opioids [89]. PAP therapy use may be additionally affected by opioid use, as opioids may complicate underlying sleep apnea and make PAP therapy less effective [90].

## Conclusion

Our population-based study suggests a significantly increased hazard of adverse health outcomes among adults referred for sleep disorder assessment who used opioids compared to those who did not, regardless of whether they had a high probability of moderate to severe OSA. These findings highlight the importance of safe prescribing of opioids in this population. The use of opioids and OSA was associated with the greatest hazard of all-cause hospitalizations and MVC requiring hospitalization or ED visit. The interaction of opioids and OSA did not confer a synergistic risk for poor outcomes. Future studies are required to further explore individual and opioid characteristics associated with the greatest risk for adverse health outcomes.

## Supporting information

S1 Fig Cumulative incidence plot for all-cause hospitalizations. (TIF)

S2 Fig Cumulative incidence plot for ischemic heart disease-related hospitalizations. (TIF)

S3 Fig Cumulative incidence plot for all-cause emergency department visits. (TIF)

S4 Fig Cumulative incidence plot for motor vehicle collision-related emergency department visits and hospitalizations. (TIF)

S5 Fig The effects of active opioid use on all-cause mortality at different probabilities of obstructive sleep apnea expressed as adjusted hazard ratios and 95% confidence intervals. (TIF)

S6 Fig The effects of active opioid use on all-cause emergency department visits at different probabilities of obstructive sleep apnea expressed as adjusted hazard ratios and 95% confidence intervals. (TIF)

S7 Fig The effects of active opioid use on all-cause hospitalizations at different probabilities of obstructive sleep apnea expressed as adjusted hazard ratios and 95% confidence intervals. (TIF)

S8 Fig The effects of active opioid use on ischemic heart disease-related hospitalizations at different probabilities of obstructive sleep apnea expressed as adjusted hazard ratios and 95% confidence intervals. (TIF)

S9 Fig The effects of active opioid use on motor vehicle collision-related emergency department visits and hospitalizations at different probabilities of obstructive sleep apnea expressed as adjusted hazard ratios and 95% confidence intervals. (TIF)

S1 Table Details on the cohort creation and variable definitions. (DOCX)

S2 Table The effects of all variables included in the final model on outcomes of interest expressed as adjusted hazard ratios (HRs) and 95% confidence intervals (CI). (DOCX)

S3 Table The effects of active opioid use on outcomes of interest at different probabilities of obstructive sleep apnea (OSA) expressed as adjusted hazard ratios (HRs) and 95% confidence intervals (CI). (DOCX)

S1 Text Measures of biological interactions: formulas and interpretation. (DOCX)

S1 File References. (DOCX)

S1 Appendix SAS code for cohort creation. (SAS)

S2 Appendix SAS code for exposure definition. (SAS)

S3 Appendix SAS code for outcomes definition. (SAS)

S4 Appendix SAS code for covariates definition. (SAS)

S5 Appendix SAS code for models. (SAS)
