# Supplementary figures and images for: Association of obstructive sleep apnea and opioids use on adverse health outcomes: A population study of health administrative data
Source: PLoS One. 2022 Jun 28;17(6):e0269112. doi: 10.1371/journal.pone.0269112 (PMC9239451; doi:10.1371/journal.pone.0269112)

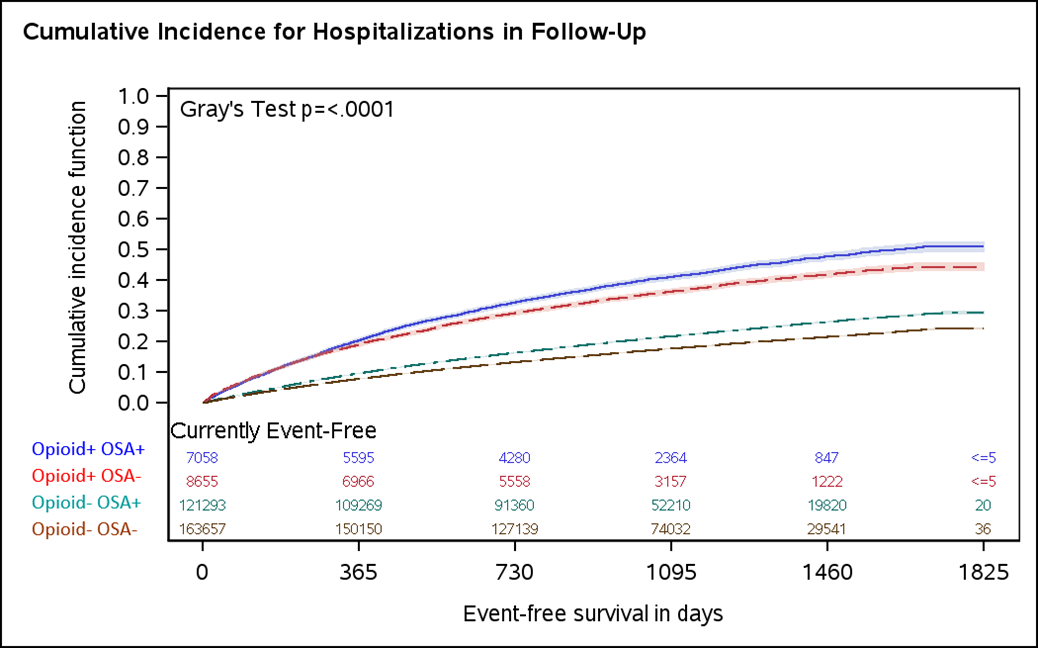

Supplement: S1 Fig — (TIF) [file pone.0269112.s001.tif]

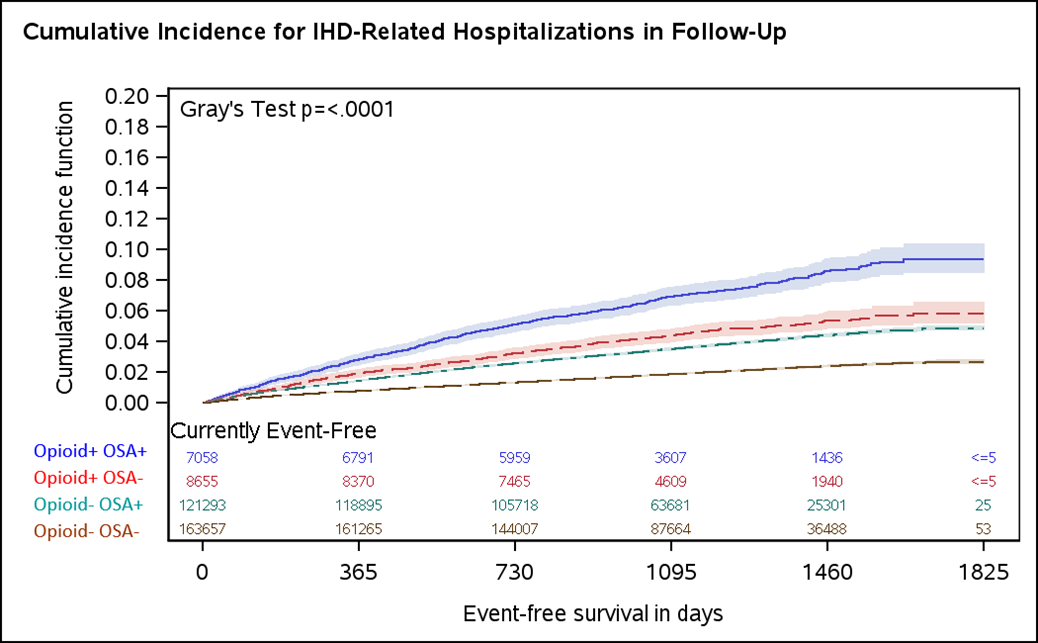

Supplement: S2 Fig — (TIF) [file pone.0269112.s002.tif]

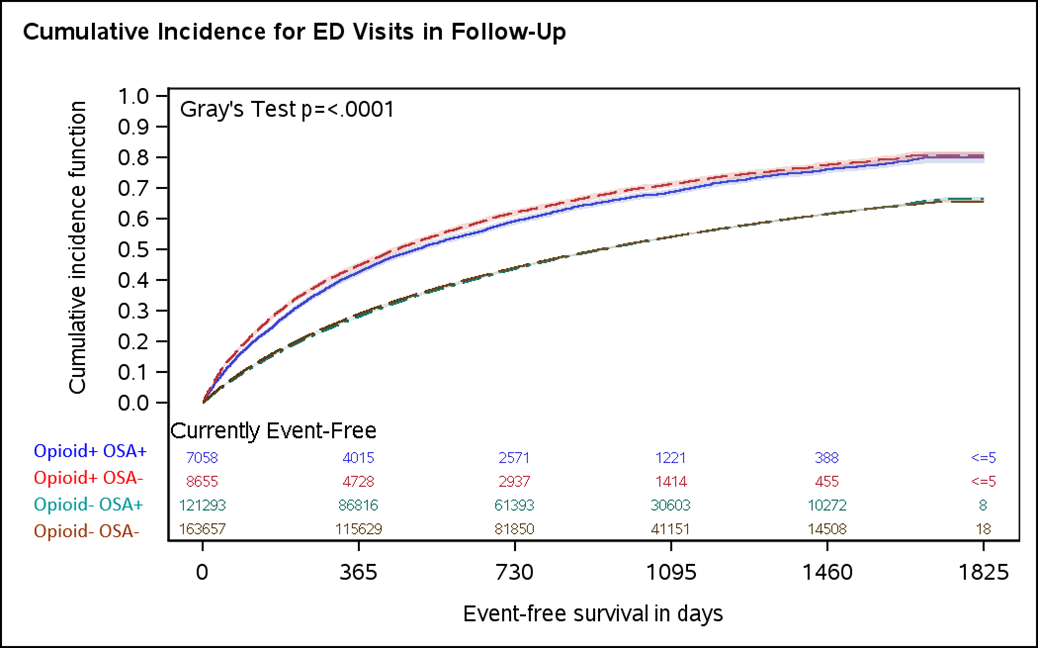

Supplement: S3 Fig — (TIF) [file pone.0269112.s003.tif]

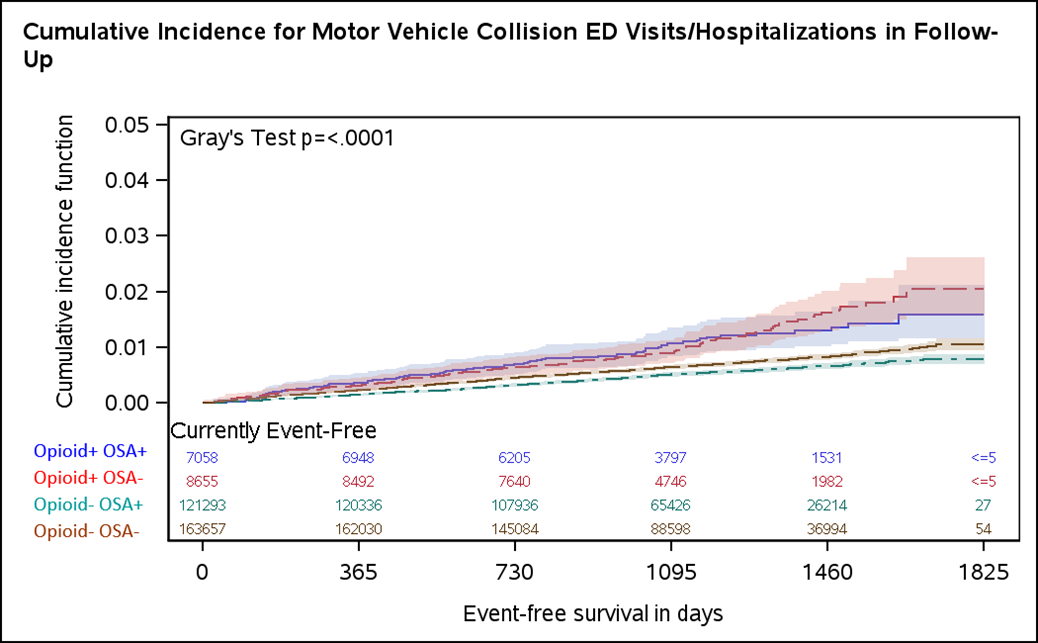

Supplement: S4 Fig — (TIF) [file pone.0269112.s004.tif]

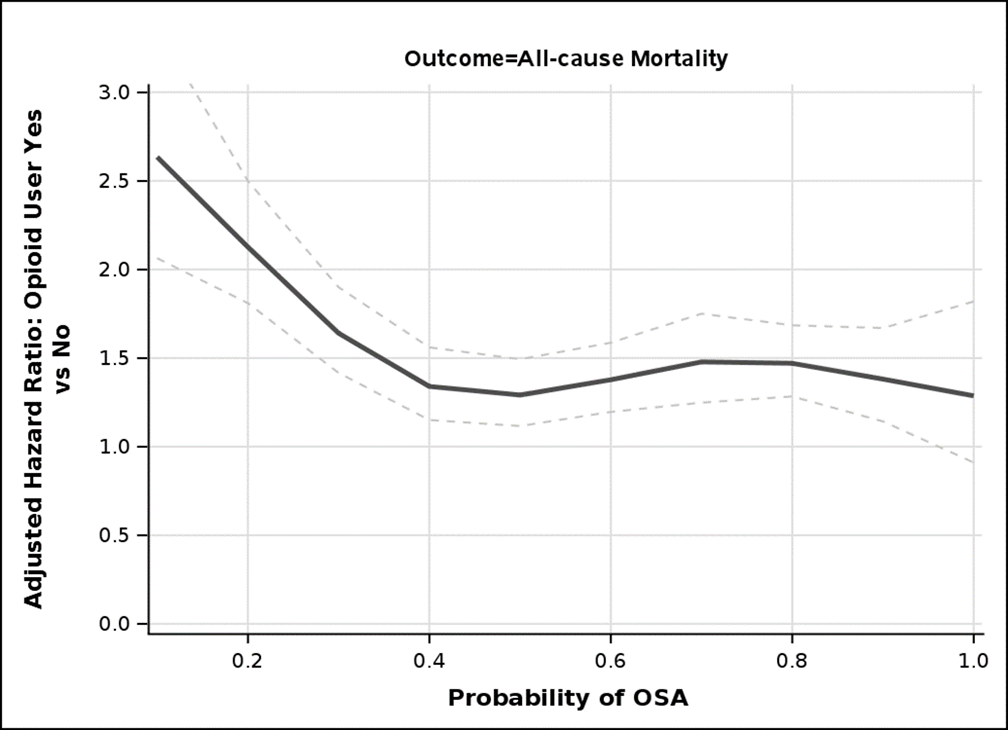

Supplement: S5 Fig — (TIF) [file pone.0269112.s005.tif]

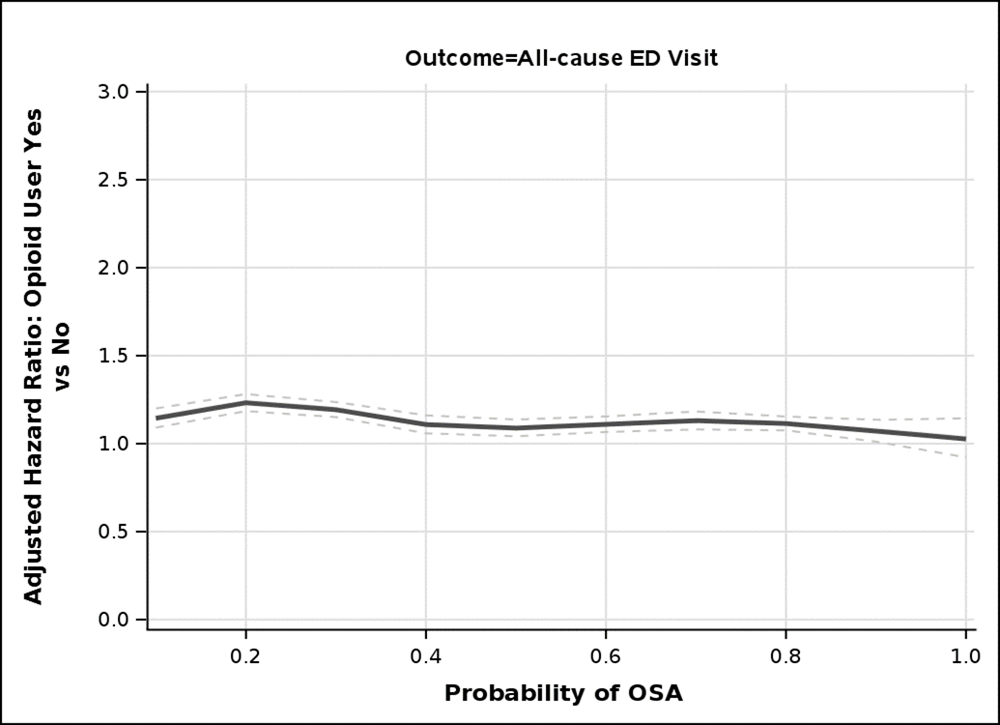

Supplement: S6 Fig — (TIF) [file pone.0269112.s006.tif]

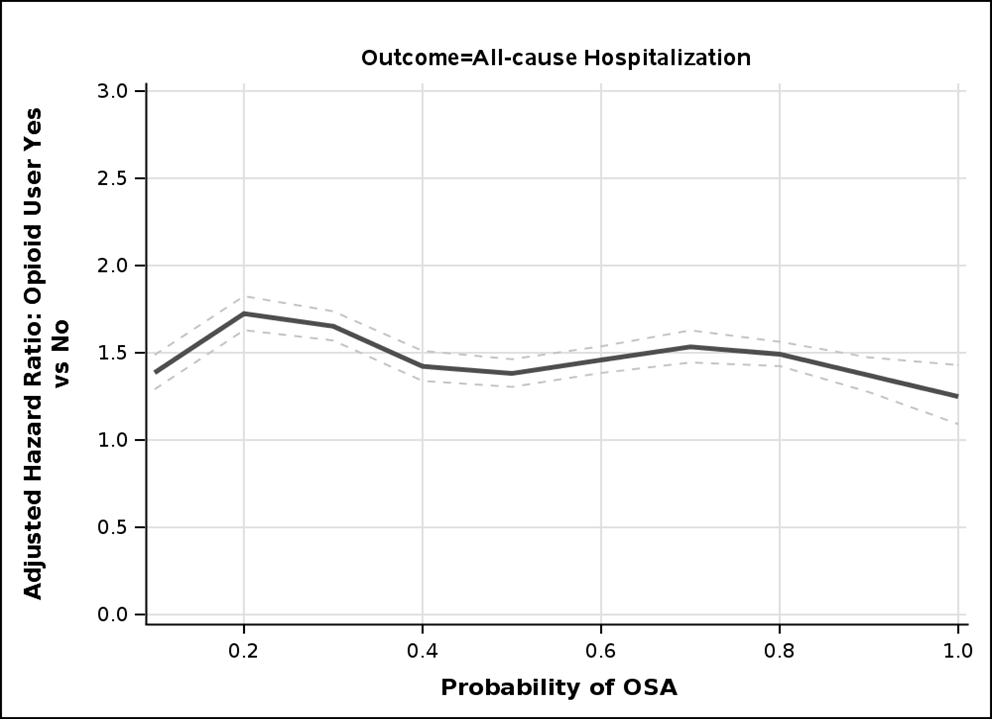

Supplement: S7 Fig — (TIF) [file pone.0269112.s007.tif]

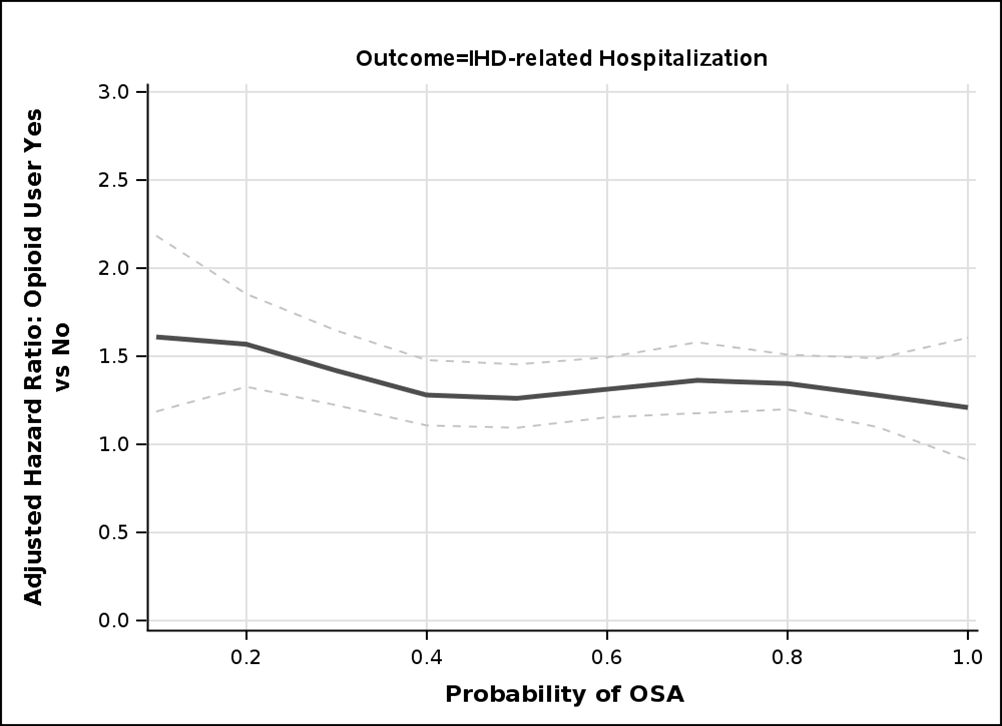

Supplement: S8 Fig — (TIF) [file pone.0269112.s008.tif]

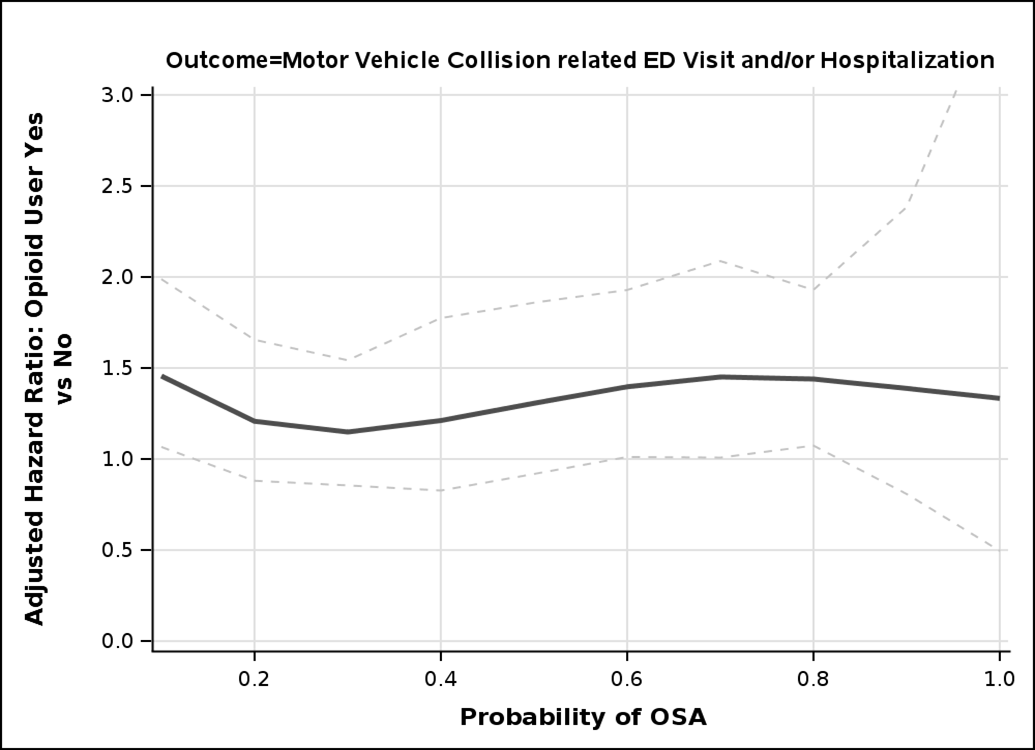

Supplement: S9 Fig — (TIF) [file pone.0269112.s009.tif]
