# Supplementary material for: Association of obstructive sleep apnea and opioids use on adverse health outcomes: A population study of health administrative data
Source: PLoS One. 2022 Jun 28;17(6):e0269112. doi: 10.1371/journal.pone.0269112 (PMC9239451; doi:10.1371/journal.pone.0269112)
Supplement: S1 Table — (DOCX) [file pone.0269112.s010.docx]

**S1 Table. Details on the cohort creation and variable definitions**

| **Cohort of Interest: individuals who underwent diagnostic sleep study, untreated for sleep disordered breathing** | **Cohort**: All adults (***18+***) who underwent a diagnostic sleep study (***index study date***) identified using the OHIP fee codes (J890, J690, J896, J696, J897, J697) from July 2013 and June 2016  **Excluded individuals**:  (1) received ***palliative care*** (based on physician service codes in OHIP and CIHI-DAD databases) in the year prior to the index date: admitted to hospital with a patient service code for palliative care (PATSERV = 58) or palliative diagnostic code (Z515) in any diagnosis field; or if a treating physician had billed OHIP for any of the following palliative care fee codes: A945, B998, C945, C882, C982, K023, W872, W882, W972 or W982; or palliative end of life homecare (service code 95 or 54) from home care delivered services OR  (2) in ***long-term care*** (LTS) in the year prior to the index date from Continuing Care Reporting System (CCRS - LTC) OR  (3) already on ***positive airway pressure (PAP) treatment*** at the index date or requested a repeat PAP prescription in the last 5 years through the Assistive Device Program Database (ADP) OR  (4) underwent a ***therapeutic sleep study*** (the OHIP fee codes: J889, J689, J895, J695) in the last 5 years preceding the index date OR  (5) were taking opioids which are rarely used and/or with not well-defined morphine equivalencies such as intranasal, injectable, or rectal suppositories opioids at the index date as well as individuals who were taking fentanyl tablets prior to the index date as those individuals may represent a unique population which is not in the focus of our study OR  (6) Missing age or gender OR  (7) Uninsured |  |
| --- | --- | --- |
|  |  |  |
| **Exposures** | |  |
| **Opioid usage at the index date** | |  |
| **Opioids of interest** (the Narcotic Monitoring System [NMS] database) | Individuals on opioids will be defined through the NMS database by dispensing of **oral/transdermal** opioids between July 2012 and March 2018.  At least one opioid prescription over the study period, including **oral formulations** of morphine, codeine, oxycodone, meperidine, hydromorphone, and pentazocine, tramadol, tapentadol, opium (miscellaneous opioids) as well as **transdermal** fentanyl and buprenorphine patches. |  |
| **Opioid Exposure** | Active opioid prescription: An opioid Rx that overlaps with the index date (i.e., date of dispensing is < index date and date of dispensing + days supply is >index date) |  |
| **The average daily dose (Morphine equivalent doses, MME) for active and recent opioid prescriptions** | The average daily dose was calculated as the dose (in milligrams) divided by the number of days’ supply for which the prescription was written, converted to morphine equivalents using morphine equivalence ratios used by the Canadian National Opioid Use Guideline Group. When multiple concurrent opioid prescriptions were identified, the total average daily dose was defined as the sum of the average daily dose of all prescriptions overlapping the patient’s index date. |  |
|  |  |  |
| **Probability of Moderate to severe obstructive sleep apnea (OSA)** using previously internally and externally validated case-ascertainment model against a diagnostic sleep study (gold standard).^5^ This model contained six variables in relation to an index sleep study: an outpatient visit for OSA from a specialist physician, a repeated sleep study and a positive airway pressure (PAP) treatment claim within 1 year of the index sleep study, patient sex and age at the index sleep study and hospitalizations with hypertension in the last 3 years prior to the sleep study.^5^ | |  |
| **Primary: Individuals with an estimated probability of 0.5 or greater** | On the external cohort, this definition yielded a sensitivity of 59% (95% CI: 58–60), specificity of 87% (95% CI: 0.87–0.88), a positive predictive value of 0.79 (95% CI: 0.78–0.80) and negative predictive value of 0.73 (95% CI: 0.72–0.74) to identify individuals ***with an estimated probability of 0.5 or greater*** of moderate to severe OSA.^5^ |  |
| **Secondary: Probability as a continuous variable ranging between 0 and 1 by 0.1 units** | - 0.1: Sen=0.98; Sp=0.11; LR+=1.10; LR-= 0.17 - 0.2: Sen=0.92; Sp=0.37; LR+=1.46; LR-= 0.22 - 0.3: Sen=0.80; Sp=0.65; LR+=2.28; LR-= 0.31 - 0.4: Sen=0.68; Sp=0.80; LR+=3.42; LR-= 0.40 - 0.5: Sen=0.58; Sp=0.88; LR+=4.78; LR-= 0.47 - 0.6: Sen=0.47; Sp=0.92; LR+=5.88; LR-= 0.57 - 0.7: Sen=0.36; Sp=0.95; LR+=7.23; LR-= 0.67 - 0.8: Sen=0.23; Sp=0.97; LR+=8.73; LR-= 0.79 - 0.9: Sen=0.04; Sp=1.00; LR+=14.44; LR-= 0.96 - 1: Sen=0.00; Sp=1.00; LR+=.; LR-= 1.00 |  |
|  | |  |
| **Outcomes: from the index date to the last date of the follow-up** (***March 31, 2018***) | | |
| **Primary outcome: All-cause mortality (from RPDB – Demographic database)** | | The date of the death from all-causes |
| **Secondary outcomes** | |  |
| **All-cause Hospitalization (from DAD database)** | | The date of inpatient hospitalization for all-causes |
| **All-cause Emergency Department Visit that does not result in a hospitalization (from NACRS database)** | | The date of emergency department visit for all-causes |
| **Ischemic Heart Disease hospitalizations (from DAD database)** | | ER visits or hospitalizations for IHD: any diagnostic type to indicate that a patient had IHD plus procedure codes for PCI and CABG (a specificity of 99.4% [95% CI 98.9–99.9%] and a sensitivity of 62.1% [95% CI 51.9–72.3%])^6^  CIHI (DAD):   - ICD-9: 410–414 - ICD-10: I20–I25   For the interventions (CCP codes):  PCI: 4802, 4803 and 4809 and the CCI codes: 1IJ50 and 1IJ57GQxx  CABG: 481 and the CCI code: 1IJ76 |
| **Motor vehicle crashes requiring hospital or emergency department visit** | | Hospitalizations and emergency department visits involving crashes in which the patient was the driver of the motor vehicle and exclude emergency department visits involving crashes in which the patient was a passenger or pedestrian^7^  ICD-10 codes:   - V20-V29 Motorcycle rider injured in transport accident - V30-V39 Occupant of three-wheeled motor vehicle injured in transport accident - V40-V49 Car occupant injured in transport accident - V50-V59 Occupant of pick-up truck or van injured in transport accident - V60-V69 Occupant of heavy transport vehicle injured in transport accident |
|  | |  |
| **Variable Definitions (Baseline Characteristics)** | | |
| **Baseline demographics** | | - Age, sex - Socioeconomic status (SES): A patient's residential neighbourhood income was defined from the Ontario Census. Ontario neighbourhoods are classified into one of the five approximately equal-sized income quintiles, ranked from poorest (Q1) to wealthiest (Q5) and shown to be related to population health status and health care utilization^8^. The neighborhood income quintiles have previously been shown to be a useful method to stratify individuals by SES and to identify related disparities in health and health care utilization^8^. Research has demonstrated that the neighborhood-level income measures may not only account for the aspects of individual-level SES, such as income and education level, but also measure contextual factors of SES, such as access to resources, availability and quality of local services, rates of crime and violence, unemployment rates, and features of the social environment (e.g., social interaction, physical activity)^9,10^. - Location of residence (urban vs. rural) |
| **Information on all medications available in NMS database one year prior to the index date** | | - being on any opioids prescribed in past year (Yes/No) - being on benzodiazepines (Yes/No) - being on barbiturates (Yes/No) - being on cannabinoids (Yes/No) - being on stimulants (Yes/No) - being on testosterone (Yes/No) |
| **Opioid use disorder** | |  |
| **Individuals who were hospitalized for opioid use disorder in the last five years preceding the index date (from CIHI)** | | Hospitalizations and/or ED visits for (ICD-10-CA codes):   - T40.0 (poisoning by opium) - T40.1 (poisoning by heroin) - T40.2 (poisoning by other opioids) - T40.3 (poisoning by methadone) - T40.4 (poisoning by other synthetic narcotics) - T40.6 (poisoning by other and unspecified narcotics)^11^ |
| **Individuals who were taking narcotics for opioid use disorder in the last year prior the index date (from NMS** **database)** | | Anyone taking orally methadone and buprenorphine (including in combination with naloxone, e.g., Suboxone) |
| **Mental and behavioural disorders due to use of opioids (from DAD, NACRS and OMHRS databases) in the last 5 years** | | (1) from DAD and NACRS (hospitalizations/ED visits):   - ICD-10: F11 (Mental and behavioural disorders due to use of opioids. Details are in the Appendix)   (2) from OMHRS:   - 304.00: Opioid dependence - 305.50: Opioid abuse |
| **Prior comorbidities: Comorbidities at index date that can also affect prescription of opioids: hypertension, diabetes, depression, psychiatric comorbidities, liver disease, asthma, COPD (including severe COPD which may require an opioid prescription), cardiovascular disorders, chronic renal disorder, prior health care utilization and surgical interventions; comorbidities associated with early mortality (e.g., cancer and being on dialysis); conditions that can contribute to sleep disordered breathing such as neuromuscular disorders and alcohol intoxication/abuse.** | | |
| **17 ICES chronic conditions at the index date (https://datadictionary.ices.on.ca/Applications/DataDictionary/Default.aspx)** | | Validated algorithms^12-20^ were used to ascertain cases of the following 8 chronic conditions:  1. Acute myocardial infarction^18^  2. Asthma^14^  3. Congestive Heart Failure^12^  4. COPD (sensitive cohort)^13^  5. Dementia^15^  6. Diabetes (the Ontario Diabetes Database)^16^  7. Hypertension (the Hypertension Database)^20^  8. Rheumatoid Arthritis (the Ontario Rheumatoid Arthritis Database)^19^  The remaining 10 chronic conditions were defined according to inpatient hospital diagnostic codes (at least 1 from DAD) or outpatient physician billing codes (at least 2 from OHIP within a 2-year period):  1. Arrhythmia  2. Coronary Heart Disease  3. IBD  4. Non-psychotic mood and anxiety disorders  5. Osteoarthritis  6. Osteoporosis  7. Other mental health conditions  8. Stroke |
| **Measure of Comorbidity** | | The Charlson comorbidity index (CCI)^21^ 2 years prior to index, aggregated, n (%):   - none (CCI score = 0) - low (score = 1) - moderate (score = 2) - high (score ≥ 3) |
| **Presumably moderate to severe COPD** | | Prevalent COPD from the ICES-derived COPD specific cohort^13^ |
| **Hospitalizations with serious liver disease** in the last 5 years prior the index date | | from DAD/SDS:   - ICD-9: 5712, 5715, 5716 - ICD-10: K703, K71.7, K74 |
| **End stage renal disease, hemodialysis** in the last 5 years prior the index date (from DAD, SDS, NACRS, and/or OHIP databases) | | - Any hospitalization or same day record from DAD, or NACRS - ICD-9: 4031, 4039, 585, V45.1 - ICD-10: I12, I13, N18.3, 18.4, 18.5, 18.6, 18.9, E08.22, E09.22, E10.22, E11.22, E13.22, Z99.2 - OHIP codes: G860, G861, G862, G863, G864, G865, G866 |
| **Cancer** | | Prevalent cancer from the Ontario Cancer Registry^22^ |
| **Number of the office visit, primary care**, in the last year prior the index date | | Obtain all OHIP records for the desired period, where visit location was in the physician office, LTC (the physician came to a long-term care facility to see the patient), or home (i.e., patient’s home) |
| **Any outpatient or inpatient surgical intervention in the last year** | | From DAD database, using intervention indicator |
| **In the last 5 years** | |  |
| **Alcohol dependence/ intoxication** (from DAD, SDS, NACRS, and/or OHIP databases) | | - Any hospitalization, ED visit or same day record from DAD, SDS or NACRS - ICD-9: 303, 3050 - ICD-10: E512, F10, G312, G621, G721, I426, K292, K70, K860, T510, X45, X65, Y15, Y573, Z502, Z714, Z721   • OHIP code: 303 |
| **Neuromuscular Disease^23^** (from DAD, OHIP databases) | | - ICD-9, ICD-10 and OHIP codes for the following conditions: Amyotrophic lateral sclerosis, Cerebral palsy, Guillain-Barre syndrome, Metabolic disorders, Multiple sclerosis, Muscular dystrophy, Myasthenia gravis, Neuromuscular disorders (other), Neuropathy, Post-polio syndrome, Spina bifida, Spinal muscular atrophy - For patients identified with OHIP dx349, including only those with subsequent or previous NMD-related ED, hospitalization visit or with subsequent or previous neurologist visit and EMG |

DAD, the Discharge Abstract Database (Canadian Institute for Health Information); NACRS, the National Ambulatory Care Reporting System Metadata (Canadian Institute for Health Information); COPD, chronic obstructive pulmonary disease; ED, emergency department; ICD, International Classification of Diseases; NMS, Narcotics Monitoring System; OHIP, the Ontario Health Insurance Plan Database; OMHRS, the Ontario Mental Health Reporting System; RPDB, the Registered Persons Database; SDS, Same Day Surgery
