# Supplementary material for: Association of obstructive sleep apnea and opioids use on adverse health outcomes: A population study of health administrative data
Source: PLoS One. 2022 Jun 28;17(6):e0269112. doi: 10.1371/journal.pone.0269112 (PMC9239451; doi:10.1371/journal.pone.0269112)
Supplement: S2 Table — (DOCX) [file pone.0269112.s011.docx]

**S2 Table. The effects of all variables included in the final model on outcomes of interest expressed as adjusted hazard ratios (HRs) and 95% confidence intervals (CI)**

|  | | **All-cause Mortality**  **(N=** **6,204)** | **All-cause ED Visit**  **(N=** **166,997)** | **All-cause Hospitalization**  **(N=** **63,208)** | **IHD-related Hospitalization**  **(N=** **8,549)** | **MVC related ED Visit or Hospitalization**  **(N=** **1,942)** |
| --- | --- | --- | --- | --- | --- | --- |
| Opioid+ OSA+ | | 1.14 (1.02-1.27) | 1.11 (1.08-1.15) | 1.55 (1.49-1.61) | 1.33 (1.21-1.47) | 1.39 (1.09-1.77) |
| Opioid+ OSA- | | 1.75 (1.57-1.94) | 1.17 (1.14-1.20) | 1.53 (1.47-1.59) | 1.38 (1.24-1.54) | 1.26 (1.02-1.55) |
| Opioid- OSA+ | | 0.80 (0.75-0.84) | 1.01 (1.00-1.02) | 1.06 (1.05-1.08) | 0.99 (0.95-1.04) | 0.95 (0.86-1.06) |
| Opioid- OSA- | | Reference | Reference | Reference | Reference | Reference |
|  | |  |  |  |  |  |
| **Demographics at the index date (the date of the sleep study)** | | | | | | |
| Age: per one year increase | | 1.07 (1.06-1.07) | 0.99 (0.99-0.99) | 1.01 (1.01-1.01) | 1.04 (1.03-1.04) | 0.98 (0.97-0.98) |
| Sex: Female vs Male | | 0.71 (0.67-0.75) | 1.17 (1.16-1.19) | 1.29 (1.27-1.31) | 0.58 (0.55-0.61) | 1.37 (1.25-1.51) |
| Neighbourhood Income Quintile | 1 (lowest) | Reference | Reference | Reference | Reference | Reference |
|  | 2 | 0.92 (0.85-0.99) | 0.93 (0.91-0.94) | 0.95 (0.93-0.97) | 0.87 (0.82-0.93) | 0.89 (0.78-1.01) |
|  | 3 | 0.83 (0.77-0.89) | 0.89 (0.88-0.90) | 0.91 (0.89-0.93) | 0.88 (0.83-0.94) | 0.84 (0.73-0.96) |
|  | 4 | 0.83 (0.77-0.90) | 0.86 (0.84-0.87) | 0.90 (0.87-0.92) | 0.85 (0.79-0.91) | 0.78 (0.68-0.89) |
|  | 5 (highest) | 0.81 (0.75-0.87) | 0.79 (0.78-0.81) | 0.88 (0.86-0.90) | 0.92 (0.86-0.98) | 0.63 (0.54-0.73) |
| Rurality: No vs Yes | | 0.83 (0.78-0.89) | 0.61 (0.60-0.62) | 0.85 (0.83-0.87) | 0.90 (0.84-0.96) | 0.82 (0.72-0.94) |
|  | |  |  |  |  |  |
| **Primary health care exposure and surgical interventions in the last year** | | | | | | |
| Number of Primary Care Visits, per unit increase | | 1.01 (1.00-1.01) | 1.01 (1.01-1.01) | 1.01 (1.01-1.01) | 1.00 (1.00-1.00) | 1.02 (1.01-1.02) |
| Surgery/Intervention Indicator, per unit increase | | 0.95 (0.88-1.03) | 1.10 (1.07-1.13) | 1.30 (1.26-1.34) | 1.18 (1.11-1.26) | 1.01 (0.81-1.27) |
|  | |  |  |  |  |  |
| **Substances’ use** | | | | | | |
| Alcohol Dependence/intoxication (last 5 years) | | 2.02 (1.81-2.25) | 1.30 (1.27-1.34) | 1.41 (1.35-1.47) | 1.22 (1.08-1.38) | 1.41 (1.12-1.79) |
| Benzodiazepine Dispensed (last year) | | 1.19 (1.11-1.26) | 1.18 (1.17-1.20) | 1.13 (1.10-1.15) | 1.11 (1.05-1.18) | 1.12 (0.99-1.26) |
|  | |  |  |  |  |  |
| **Comorbidities** | | | | | | |
| Charlson Comorbidity Index (last year), per unit increase | | 1.28 (1.26-1.31) | 1.09 (1.08-1.10) | 1.18 (1.17-1.20) | 1.21 (1.18-1.23) | 1.06 (0.98-1.16) |
| Prevalent Chronic heart failure | | 2.41 (2.25-2.58) | 1.16 (1.13-1.19) | 1.58 (1.53-1.63) | 1.70 (1.60-1.80) | 1.11 (0.86-1.44) |
| Prevalent Chronic obstructive pulmonary disease | | 1.93 (1.81-2.06) | 1.31 (1.29-1.34) | 1.56 (1.51-1.60) | 1.35 (1.27-1.44) | 1.03 (0.82-1.29) |
| Prevalent Coronary artery disease | | 1.09 (1.02-1.15) | 1.24 (1.22-1.26) | 1.27 (1.24-1.30) | 3.71 (3.52-3.90) | 1.28 (1.09-1.50) |
| Prevalent Arrythmias | | 1.13 (1.06-1.20) | 1.26 (1.24-1.28) | 1.28 (1.25-1.32) | 0.92 (0.87-0.98) | 1.01 (0.83-1.21) |
| Prevalent Diabetes | | 1.26 (1.19-1.33) | 1.10 (1.09-1.12) | 1.26 (1.24-1.29) | 1.42 (1.36-1.49) | 1.27 (1.12-1.43) |
| Prevalent Hypertension | | 1.25 (1.17-1.34) | 1.08 (1.07-1.10) | 1.27 (1.25-1.30) | 1.75 (1.65-1.86) | 1.03 (0.92-1.15) |
| Non-psychotic Mood and Anxiety Disorders | | 1.06 (1.00-1.13) | 1.17 (1.16-1.18) | 1.04 (1.02-1.06) | 1.00 (0.95-1.06) | 1.19 (1.08-1.32) |
| (Other) Mental Health Conditions prevalent | | 0.95 (0.89-1.01) | 1.01 (0.99-1.02) | 0.94 (0.92-0.96) | 0.88 (0.84-0.93) | 0.98 (0.89-1.09) |
| Prevalent Cancer | | 1.63 (1.53-1.74) | 1.12 (1.10-1.15) | 1.29 (1.26-1.33) | 0.98 (0.91-1.04) | 1.04 (0.84-1.28) |
| Prevalent Osteoarthritis | | 0.87 (0.83-0.92) | 1.22 (1.20-1.23) | 1.21 (1.19-1.23) | 1.07 (1.02-1.12) | 1.27 (1.15-1.40) |
| Neuromuscular Disease (last 5 years) | | 1.44 (1.32-1.56) | 1.18 (1.16-1.21) | 1.18 (1.15-1.22) | 1.09 (1.01-1.18) | 1.26 (1.06-1.50) |

OSA, obstructive sleep apnea
