# Supplementary material for: Association of obstructive sleep apnea and opioids use on adverse health outcomes: A population study of health administrative data
Source: PLoS One. 2022 Jun 28;17(6):e0269112. doi: 10.1371/journal.pone.0269112 (PMC9239451; doi:10.1371/journal.pone.0269112)
Supplement: S3 Table — (DOCX) [file pone.0269112.s012.docx]

**S3 Table.** **The effects of active opioid use on outcomes of interest at different probabilities of obstructive sleep apnea (OSA) expressed as adjusted hazard ratios (HRs) and 95% confidence intervals (CI).**

| **Probability of moderate to severe OSA** (from 0 to 1 by 0.1 units) | **All-cause Mortality** | **All-cause ED Visit** | **All-cause Hospitalization** | **IHD-related Hospitalization** | **Motor Vehicle Collision related ED Visit and/or Hospitalization** |
| --- | --- | --- | --- | --- | --- |
| 0.1 | 2.64 (2.06-3.36) | 1.14 (1.09-1.20) | 1.39 (1.29-1.49) | 1.61 (1.19-2.18) | 1.46 (1.07-1.99) |
| 0.2 | 2.13 (1.81-2.50) | 1.23 (1.19-1.28) | 1.72 (1.63-1.82) | 1.57 (1.33-1.85) | 1.21 (0.88-1.66) |
| 0.3 | 1.64 (1.42-1.90) | 1.19 (1.15-1.24) | 1.65 (1.57-1.74) | 1.42 (1.22-1.65) | 1.15 (0.86-1.54) |
| 0.4 | 1.34 (1.15-1.56) | 1.11 (1.06-1.16) | 1.42 (1.34-1.51) | 1.28 (1.11-1.48) | 1.21 (0.83-1.77) |
| 0.5 | 1.29 (1.12-1.49) | 1.09 (1.04-1.14) | 1.38 (1.30-1.46) | 1.26 (1.09-1.45) | 1.31 (0.92-1.86) |
| 0.6 | 1.38 (1.20-1.59) | 1.11 (1.07-1.16) | 1.46 (1.38-1.54) | 1.31 (1.15-1.49) | 1.40 (1.01-1.93) |
| 0.7 | 1.48 (1.25-1.75) | 1.13 (1.08-1.18) | 1.53 (1.44-1.63) | 1.36 (1.18-1.58) | 1.45 (1.01-2.09) |
| 0.8 | 1.47 (1.28-1.69) | 1.11 (1.08-1.15) | 1.49 (1.42-1.56) | 1.35 (1.20-1.51) | 1.44 (1.07-1.93) |
| 0.9 | 1.38 (1.14-1.67) | 1.07 (1.01-1.14) | 1.37 (1.27-1.47) | 1.28 (1.10-1.49) | 1.39 (0.81-2.38) |
| 1 | 1.29 (0.91-1.82) | 1.03 (0.92-1.14) | 1.25 (1.09-1.43) | 1.21 (0.91-1.60) | 1.33 (0.50-3.59) |
| **P value for the interaction** | <0.001 | <0.001 | <0.001 | 0.3771 | 0.819 |

ED, emergency department; IHD, ischemic heart disease; OSA, obstructive sleep apnea
