# Supplementary material for: Association of obstructive sleep apnea and opioids use on adverse health outcomes: A population study of health administrative data
Source: PLoS One. 2022 Jun 28;17(6):e0269112. doi: 10.1371/journal.pone.0269112 (PMC9239451; doi:10.1371/journal.pone.0269112)
Supplement: S1 Text — (DOCX) [file pone.0269112.s013.docx]

**S1 Text. Measures of biological interactions: formulas and interpretation**

Rothman presents three measures of biological interaction on the additive scale: (i) the relative excess risk due to interaction (RERI), (ii) the attributable proportion due to interaction (AP), and (iii) the synergy index (S).^1^

These measures were defined as follows:^1^

For two dichotomous factors (e.g., opioid use and moderate to severe obstructive sleep apnea [OSA]): RR11 is the relative risk of the outcome if both factors are present (Opioid+ OSA+), RR10 is the relative risk of the outcome if the first factor is present but second factor is absent (Opioid+ OSA–), RR01 is the relative risk of the outcome if the first factor is absent but second factor is present (Opioid– OSA+).

**(i) RERI = RR11 - RR10 - RR01 + 1**

Interpretation: RERI can range from − infinity to + infinity. RERI = 0 means no interaction or exactly additivity; RERI > 0 means positive interaction or more than additivity; RERI < 0 means negative interaction or less than additivity. However, as suggested by Li & Chambless in the proportional hazards model *RERI_HR_* > 0 means positive interaction only under an assumption that both exposures have positive monotonic effects on the outcome; to draw the same conclusion without monotonicity assumptions requires *RERI_HR_* > 1.^2^

**(ii) AP = RERI/RR11**

Interpretation: AP can range from −1 to +1. AP = 0 means no interaction or exactly additivity; AP > 0 means positive interaction or more than additivity; AP < 0 means negative interaction or less than additivity.

**(iii) S = [RR11 – 1]/ [(RR10- 1) + (RR01- 1)]**

Interpretation: S can range from 0 to infinity. S = 1 means no interaction or exactly additivity; S > 1 means positive interaction or more than additivity; S < 1 means negative interaction or less than additivity.

We calculated the three measures of interaction on an additive scale (RERI, AP, and S) and their 95% confidence intervals using the delta method,^3^ assuming that the hazard ratios calculated using the Cox regression model approximated relative risks; SAS code and an Excel sheet provided by Andersson and co-authors were used.^4^
