# Supplementary material for: Association of obstructive sleep apnea and opioids use on adverse health outcomes: A population study of health administrative data
Source: PLoS One. 2022 Jun 28;17(6):e0269112. doi: 10.1371/journal.pone.0269112 (PMC9239451; doi:10.1371/journal.pone.0269112)
Supplement: S1 File — (DOCX) [file pone.0269112.s014.docx]

**References**:

1. Rothman KJ. *Modern epidemiology.* 1st ed. Boston ; Toronto: Little, Brown and Company; 1986.

2. Li R, Chambless L. Test for additive interaction in proportional hazards models. *Annals of epidemiology.* 2007;17(3):227-236.

3. Hosmer DW, Lemeshow S. Confidence interval estimation of interaction. *Epidemiology.* 1992;3(5):452-456.

4. Andersson T, Alfredsson L, Kallberg H, Zdravkovic S, Ahlbom A. Calculating measures of biological interaction. *European journal of epidemiology.* 2005;20(7):575-579.

5. Kendzerska T, van Walraven C, McIsaac DI, et al. Case-Ascertainment Models to Identify Adults with Obstructive Sleep Apnea Using Health Administrative Data: Internal and External Validation. *Clinical epidemiology.* 2021;13:453-467.

6. Tu K, Mitiku T, Lee DS, Guo H, Tu JV. Validation of physician billing and hospitalization data to identify patients with ischemic heart disease using data from the Electronic Medical Record Administrative data Linked Database (EMRALD). *The Canadian journal of cardiology.* 2010;26(7):e225-228.

7. Redelmeier DA, Yarnell CJ, Thiruchelvam D, Tibshirani RJ. Physicians' warnings for unfit drivers and the risk of trauma from road crashes. *The New England journal of medicine.* 2012;367(13):1228-1236.

8. Wilkins R, Tjepkema M, Mustard C, Choiniere R. The Canadian census mortality follow-up study, 1991 through 2001. *Health reports / Statistics Canada, Canadian Centre for Health Information = Rapports sur la sante / Statistique Canada, Centre canadien d'information sur la sante.* 2008;19(3):25-43.

9. Pampalon R, Hamel D, Gamache P. A comparison of individual and area-based socio-economic data for monitoring social inequalities in health. *Health reports / Statistics Canada, Canadian Centre for Health Information = Rapports sur la sante / Statistique Canada, Centre canadien d'information sur la sante.* 2009;20(4):85-94.

10. Holowaty EJ, Norwood TA, Wanigaratne S, Abellan JJ, Beale L. Feasibility and utility of mapping disease risk at the neighbourhood level within a Canadian public health unit: an ecological study. *International journal of health geographics.* 2010;9:21.

11. *Quality Standards. Opioid Use Disorder Measurement Guide.* 2018.

12. Schultz SE, Rothwell DM, Chen Z, Tu K. Identifying cases of congestive heart failure from administrative data: a validation study using primary care patient records. *Chronic diseases and injuries in Canada.* 2013;33(3):160-166.

13. Gershon AS, Wang C, Guan J, Vasilevska-Ristovska J, Cicutto L, To T. Identifying individuals with physcian diagnosed COPD in health administrative databases. *COPD.* 2009;6(5):388-394.

14. Gershon A WC, Vasilevska-Ristovska J, Guan J, Cicutto L, To T. Identifying patients diagnosed with asthma using health administrative data. *Canadian Respiratory Journal.* 2009;16:183–188.

15. Jaakkimainen RL, Bronskill SE, Tierney MC, et al. Identification of Physician-Diagnosed Alzheimer's Disease and Related Dementias in Population-Based Administrative Data: A Validation Study Using Family Physicians' Electronic Medical Records. *J Alzheimers Dis.* 2016;54(1):337-349.

16. Hux JE, Ivis F, Flintoft V, Bica A. Diabetes in Ontario: determination of prevalence and incidence using a validated administrative data algorithm. *Diabetes Care.* 2002;25(3):512-516.

17. Tu K, Campbell NR, Chen ZL, Cauch-Dudek KJ, McAlister FA. Accuracy of administrative databases in identifying patients with hypertension. *Open Med.* 2007;1(1):e18-26.

18. Austin PC, Daly PA, Tu JV. A multicenter study of the coding accuracy of hospital discharge administrative data for patients admitted to cardiac care units in Ontario. *American heart journal.* 2002;144(2):290-296.

19. Widdifield J, Bernatsky S, Paterson JM, et al. Accuracy of Canadian health administrative databases in identifying patients with rheumatoid arthritis: a validation study using the medical records of rheumatologists. *Arthritis care & research.* 2013;65(10):1582-1591.

20. Public Health Agency of Canada. Report from the Canadian Chronic Disease Surveillance System hypertension in Canada, 2010. [Ottawa]: Public Health Agency of Canada; 2010: https://www.canada.ca/en/public-health/services/chronic-diseases/cardiovascular-disease/report-canadian-chronic-disease-surveillance-system-hypertension-canada-2010.html [Access Apr 2, 2022]

21. Sundararajan V, Henderson T, Perry C, Muggivan A, Quan H, Ghali WA. New ICD-10 version of the Charlson comorbidity index predicted in-hospital mortality. *Journal of clinical epidemiology.* 2004;57(12):1288-1294.

22. McLaughlin JR, Kreiger N, Marrett LD, Holowaty EJ. Cancer incidence registration and trends in Ontario. *Eur J Cancer.* 1991;27(11):1520-1524.

23. Rose L, McKim D, Leasa D, et al. Trends in incidence, prevalence, and mortality of neuromuscular disease in Ontario, Canada: A population-based retrospective cohort study (2003-2014). *PLoS One.* 2019;14(3):e0210574.
